# Methyltransferase-Deficient Avian Flaviviruses Are Attenuated Due to Suppression of Viral RNA Translation and Induction of a Higher Innate Immunity

**DOI:** 10.3389/fimmu.2021.751688

**Published:** 2021-10-06

**Authors:** Xuedong Wu, Yuetian Zhang, Mingshu Wang, Shun Chen, Mafeng Liu, Dekang Zhu, Xinxin Zhao, Ying Wu, Qiao Yang, Shaqiu Zhang, Juan Huang, Xumin Ou, Ling Zhang, Yunya Liu, Yanling Yu, Qun Gao, Sai Mao, Di Sun, Bin Tian, Zhongqiong Yin, Bo Jing, Anchun Cheng, Renyong Jia

**Affiliations:** ^1^ Research Centre of Avian Disease, College of Veterinary Medicine of Sichuan Agricultural University, Chengdu, China; ^2^ Institute of Preventive Veterinary Medicine, Sichuan Agricultural University, Chengdu, China; ^3^ Key Laboratory of Animal Disease and Human Health of Sichuan Province, Sichuan Agricultural University, Chengdu, China

**Keywords:** Tembusu virus, methyltransferase, attenuated, translation, innate immunity

## Abstract

The 5’ end of the flavivirus genome contains a type 1 cap structure formed by sequential N-7 and 2’-O methylations by viral methyltransferase (MTase). Cap methylation of flavivirus genome is an essential structural modification to ensure the normal proliferation of the virus. Tembusu virus (TMUV) (genus *Flavivirus*) is a causative agent of duck egg drop syndrome and has zoonotic potential. Here, we identified the *in vitro* activity of TMUV MTase and determined the effect of K61-D146-K182-E218 enzymatic tetrad on N-7 and 2’-O methylation. The entire K61-D146-K182-E218 motif is essential for 2’-O MTase activity, whereas N-7 MTase activity requires only D146. To investigate its phenotype, the single point mutation (K61A, D146A, K182A or E218A) was introduced into TMUV replicon (pCMV-Rep-NanoLuc) and TMUV infectious cDNA clone (pACYC-TMUV). K-D-K-E mutations reduced the replication ability of replicon. K61A, K182A and E218A viruses were genetically stable, whereas D146A virus was unstable and reverted to WT virus. Mutant viruses were replication and virulence impaired, showing reduced growth and attenuated cytopathic effects and reduced mortality of duck embryos. Molecular mechanism studies showed that the translation efficiency of mutant viruses was inhibited and a higher host innate immunity was induced. Furthermore, we found that the translation inhibition of MTase-deficient viruses was caused by a defect in N-7 methylation, whereas the absence of 2’-O methylation did not affect viral translation. Taken together, our data validate the debilitating mechanism of MTase-deficient avian flavivirus and reveal an important role for cap-methylation in viral translation, proliferation, and escape from innate immunity.

## Introduction

Tembusu virus (TMUV) is an emerging and recurrent flavivirus that mainly causes decreased egg laying rate and neuroinflammation in laying ducks and has the potential to become a zoonotic pathogen (1, 2). TMUV belongs to the family Flaviridae, the genus Flaviridae that includes other mosquito-borne human or animal pathogens such as dengue virus (DENV), West Nile virus (WNV), Zika virus (ZIKV), yellow fever virus (YFV), and Japanese encephalitis virus (JEV), etc. TMUV was first isolated from *Culex tritaeniorhynchus* mosquitoes in Kuala Lumpur, Malaysia in 1955 (3). Over nearly 60 years of evolution, Tembusu virus has adapted to ducks. Since the first outbreak of egg drop syndrome and neuroinflammation caused by TMUV in duck farms of China in 2010, this virus has been one of major pathogen in the duck industry of China, impacting the duck industry and causing huge economic losses (4). Researchers have detected TMUV from ducks, chickens, geese, sparrows, and mosquitoes. The territorial scope of the virus has expanded, requiring strict bio-security measures or a multivalent vaccine to control its spread (5). Recent study has reported that some people without the history of contact with ducks displayed high neutralizing antibody titres to TMUV (6). Therefore, the mode of transmission of the virus from ducks or other possible hosts to humans should be explored further.

Like other flaviviruses, TMUV genome is an approximately 11-kb-long single-stranded positive-sense RNA with a 5’ type I cap structure, followed by the strictly conserved dinucleotide sequence “AG”: 5’-m7GpppAm-G-3’ (7). The genome comprises a single large open reading frame (ORF) flanked by 5’ untranslated region (UTR) and 3’UTR and encoding a polyprotein that is processed into 3 structural proteins (C, PrM, E) and 7 non-structural proteins (NS1, NS2A, NS2B, NS3, NS4A, NS4B and NS5) (8). During virus replication, 7 non-structural proteins and host cell proteins assemble within vesicle composed of endoplasmic reticulum to form replication complex that regulates viral RNA synthesis (9, 10). The genomic (positive-sense) RNA is first used as a template to synthesize a complementary negative-sense RNA by NS5 polymerase. The nascent negative-sense RNA product exists as dsRNA, base-paired with the positive-sense RNA, and then serves as the template to generate positive stranded RNA (11). Following RNA synthesis, NS3 protein functions as helicases to unwind positive and negative double stranded RNA intermediate. The nascent positive-strand RNA synthesized on the negative RNA template displaces a pre-existing positive-strand RNA and is released as a dsRNA product. The free positive RNA is capped and methylated by NS5 methyltransferase (MTase) to form a type 1 cap structure at the 5’ end. Then, newly copied mature positive stranded RNA molecules are either recycled for replication and translation or alternatively, extruded from the vesicle for packaging into nascent virions (11).

Capping of the 5’ end of flavivirus RNA genome is an essential structural modification that limits RNA degradation by 5’-3’ exoribonucleases, conceals the viral genome from host immune sensors (12, 13), and ensures normal infection and proliferation of virus (14). It is formed by the following three successive enzymatic reactions. (I) The RNA triphosphatase of NS3 helicase hydrolyzes the γ-phosphate group of the pppAG-RNA to produce a diphosphate RNA (ppAG-RNA), (II) The guanylyltransferase located in NS5 methyltransferase region catalyzes GTP to form a covalently linked GMP-enzyme intermediate, and then transfers GMP molecule to ppAG-RNA, generating methylated precursor RNA (GpppAG-RNA), (III) GpppAG-RNA is then sequentially methylated by N-7 and 2’-O MTase according to the following scheme: GpppAG-RNA→m7GpppAG-RNA (cap 0)→m7GpppAmG-RNA (cap 1), with S-adenosyl-L-methionine (SAM) as the methyl donor (15–17). Both the N-7 and 2’-O methylation are performed by the same MTase domain located at the N terminus of NS5. N-7 methylation of the 5’-guanosine is essential for viral infection (18, 19), whereas 2’-O methylation is required for evasion of recognition by host immune sensors, notably RIG-I (retinoic acid-inducible gene I) (13, 20, 21), MDA5 (melanoma differentiation-associated protein 5) (12), and IFIT1 or IFIT5 (IFN-induced protein with tetratricopeptide repeats-1 or -5) (22–24). Among them, RIG-I recognizes short double-stranded cap0 RNA. MDA5 recognizes long double-stranded cap0 RNA. whereas IFITs recognize single-stranded cap0 RNA regardless of the length.

Crystal structures of several flavivirus MTases have been reported, such as ZIKV (25), WNV (19), DENV (26). All flavivirus MTases have a conserved K-D-K-E catalytic tetrad (NS5-K61-D146-K182-E218), which has been extensively studied. A D146A mutation that leads to defect in N-7 methylation prevents infection/propagation of DENV and WNV (19, 27), whereas K61A, K182A or E218A single mutation that completely abolishes 2’-O methylation attenuates the virus and is a basis for vaccine development (28–30). In addition, MTase has become an attractive target for the development of antiviral drugs and inhibitors (31–33). To help guide the development of vaccine and effective drugs against TMUV, we identified the *in vitro* activity of TMUV MTase and determined effect of K-D-K-E catalytic tetrad on N-7 and 2’-O methylation. The single point mutation (K61A, D146A, K182A or E218A) was introduced into TMUV replicon and recombinant TMUV infectious cDNA clone to investigate its phenotype. Further, we explored the mechanism of virus attenuation caused by these mutations. This study provides a scientific basis for the development of live attenuated vaccine and effective drugs against TMUV.

## Materials and Methods

### Viruses, Cells and Antibodies

The clinical TMUV strain CQW1 (NCBI accession no. KM233707.1) was isolated from Cherry Valley ducks in Southwest China (8). Duck embryo fibroblast (DEF) cells isolated from 9-days-old healthy duck embryo were cultured in DMEM supplemented with 10% new-born calf serum (NBCS) at 37°C with 5% CO_2_. Healthy duck embryos were purchased from a farm in Chengdu City, China. BHK-21 cells were cultured in DMEM supplemented with 10% fetal bovine serum (FBS) at 37°C with 5% CO_2_. Mouse monoclonal antibody against envelope (E) protein was prepared by our laboratory (unpublished). Fluorescein (FITC) Conjugate Goat Anti-Mouse IgG (H+L) was purchased from invitrogen.

### Recombinant MTase Preparation and Methylation Assays

TMUV NS5-MTase (1–300) from the CQW1 strain was cloned, expressed and purified from *Escherichia coli* Rosetta 2(DE3) pLysS cells with an N-terminal His tag as described previously (28). A *Fast* site directed Mutagenesis System (TransGen, China) was used to engineer an alanine mutation for K61-D146-K182-E218 motif of the TMUV MTase. Mutant primers were listed in **Supplementary Table S1**. *In vitro* RNA transcription and the RNA capping were performed as reported before (27). ^32^P-labeled RNA substrates, G*pppAG- and m7G*pppAG-RNA (representing the first 192 nucleotides of the 5’ end of the TMUV genome; the asterisk indicates that the following phosphate is ^32^P-labeled) were prepared for the MTase assay. Then, N-7 and 2’-O methylation assays were performed as described previously (28). The only difference was that the radioactive cap structure on TLC plates was quantified by a sodium iodide radioactivity detector.

### Construction and Characterization of Mutant Replicon

K61A-D146A-K182A-E218A mutant replicon plasmids were constructed based on a wild-type (WT) TMUV replicon with CMV promoter (34). A *Fast* sitedirected Mutagenesis System (TransGen, China) was used to engineer an alanine mutation for K61-D146-K182-E218 motif of the TMUV replicon. Mutant primers were the same as in **Supplementary Table S1**. To characterize the mutant replicons, BHK-21 cells were transfected with equal amounts of replicons using Lipo3000, and a Nano-Glo Luciferase Assay System (Promega) was used to detect NLuc activity of cells.

### Construction and Recovery of Mutant TMUV

K-D-K-E mutant full-length infectious cDNA clones were constructed based on mutant replicons and a recombinant TMUV infectious cDNA clone (pACYC-TMUV) (35). PCR fragments containing the K61A-D146A-K182A-E218A substitution were amplified using mutant replicon plasmids as template with primers listed in **Supplementary Table S2**. The amplified fragments were fused with pACYC-TMUV digested with EcoRv and SbfI (NEB) by homologous recombination method to obtain mutant TMUV full-length infectious cDNA clones. All cDNA plasmids were verified by DNA sequencing. The constructed plasmids were linearized by NotI endonuclease (NEB) and subjected to *in vitro* transcription using a T7 High Yield Capped RNA Transcription Kit (invitrogen). RNA transcripts were then transfected into BHK-21 cells with Lipofectamine MessengerMAX™ Reagent (invitrogen). Rescued viruses (WT, K61A, D146A, K182A and E218A) were harvested after cells developed 50% CPE or at 8 days after transfection and subjected to the following assays.

### Characterization of K-D-K-E Mutant Viruses

Indirect immunofluorescence assay (IFA) was performed to characterize the expression of TMUV E protein in BHK-21 cells transfected with equal amounts of RNA transcripts or infected with passage 1 (P1) generation virus. Briefly, infected or transfected BHK-21 cells were fixed with 4% paraformaldehyde, permeabilized with 0.25% Triton X-100 in PBS, and sealed with 5% BSA PBS, and then incubated with the mouse monoclonal antibody against E protein, followed by Fluorescein (FITC) Conjugate Goat Anti-Mouse IgG (H+L). Finally, nuclei were counterstained with 4’6-diamidino-2-phenylindole (DAPI) (Solarbio, China). The genetic stability of mutant viruses was analyzed by culturing the mutant viruses on BHK-21 cells for 10 rounds (3 to 4 days per round). After successive passage of the mutant virus on BHK-21 cells, culture fluids were collected to extract the viral genome. The specific reverse transcription primers were designed to reverse transcribed the extracted RNA into cDNA, and the whole genome sequence of each generation was verified by piecewise sequencing and splicing. The related primers were shown in **Supplementary Table S3**. The lesions caused by the mutant virus on DEF and BHK-21 cells were measured. cells were preseeded into 12-well plates and then infected with P1 WT, K61A, D146A, K182A and E218A viruses at a multiplicity of infection (MOI) of 0.01. The extent of cytopathic changes was observed daily and photographed with a microscope.

### Analysis of Growth Curve and Virulence of Mutant Viruses

Growth curves of mutant viruses in DEF and BHK-21 cells were analyzed. Briefly, cells were preseeded into 12-well plates and then infected with WT, K61A, K182A and E218A viruses at a MOI of 0.01. Culture supernatants and cells were collected at 24, 48, 72, 96 h post-infection (hpi). The culture supernatants were used to determine the virus titer by the Fluorescence Formative Unit (FFU) assays. Total RNA was extracted from cells using RNAiso Plus (TaKaRa), and the copy number of the virus was determined by absolute quantitative PCR follow the TB Green^®^ Premix Ex Taq™ II (TaKaRa) instructions.

To analyze the virulence of the mutant viruses, 9-day-old embryonated duck eggs (n=8) were infected by allantoic-cavity inoculation with 10^4^ FFU of WT, K61A, K182A or E218A virus, respectively, while the mock-infection group received 100 ul of DMEM. The mortality was then monitored daily for 2 weeks.

### Fluorescence Formative Unit Assay

BHK-21 cells were preseeded into 96-well plates and then added with 100ul of virus fluids by DMEM diluted (10–1–10–8) (n=4). At 72 h after infection, the culture medium was discarded and the cells were fixed with 4% paraformaldehyde, permeabilized with 0.25% Triton X-100 in PBS, and blocked with 5% BSA PBS, and then incubated with the mouse monoclonal antibody against E protein, followed by Fluorescein (FITC) Conjugate Goat Anti-Mouse IgG (H+L). The wells producing fluorescent spots were counted under fluorescence microscope and the fluorescence formative units were calculated according to the Reed-Muench methods.

### Quantitative Reverse Transcription PCR

Viral RNAs in culture fluids were extracted using TIANamp Virus DNA/RNA Kit (TIANGEN, China), and intracellular total RNAs were isolated using RNAiso Plus (TaKaRa). PrimeScript™ RT reagent Kit with gDNA Eraser (TaKaRa) was used to reverse transcription of purified RNA. The viral RNA copies and mRNA levels of cytokines were determined by quantitative PCR (qPCR) follow the TB Green^®^ Premix Ex Taq™ II (TaKaRa) instructions. The cycle threshold (ct) values were normalized with the mRNA level of the housekeeping gene glyceraldehyde-3-phophate dehydrogenase (GAPDH). The qPCR primers were listed in **Supplementary Table S4**.

### Quantification of Intra- and Extracellular Infectious Virions and Viral RNAs

Quantification of intra- and extracellular infectious virions and viral RNAs were carried out as modified from the work of Yang et al. (36). Briefly, at selected time points, about 2 ml of culture fluids were harvested and centrifuged at 1000g for 5 min to remove cell debris, and then divided into 1 ml/tube prior to storage at −80°C. Infected cells were treated as previously reported (36). The cell pellets were resuspended in 150 μl DMEM medium. 75 µl of the cell suspensions were centrifuged to precipitate the cells, and the precipitated cells were used to determine intracellular viral RNAs. The remaining 75 µl of the cell suspensions were lysed through a cycle of -80°C freezing and 37°C thawing. Cellular debris was removed by centrifugation and the supernatants were collected for FFU assay to determine the intracellular infectivity.

### Construction of Translation Template Plasmids, *In Vitro* Transcription, Capping and Methylation, and Analysis of Luciferase Activity

The translation template plasmids were constructed based on previously constructed monocistronic reporters (34). A modified T7 promoter sequence (5’-taatacgactcactata-3’) was placed upstream of the target sequence so that the first two bases of transcription initiation are the strictly conserved “AG” dinucleotide sequence of the flavivirus. The target sequence contains the coding sequence of RLuc flanked by the 5’UTR and 3’UTR of flavivirus (T7-5’UTR-RLuc-3’UTR), and an XhoI restriction site was placed downstream of the sequence. The translation template plasmids were linearized by XhoI endonuclease (NEB) and subjected to *in vitro* transcription using a TranscriptAid T7 high-yield transcription kit (invitrogen) to obtain uncapped RNAs (pppAG-RNA). A ScriptCap™ Cap 1 Capping System (CellScript) was used for *in vitro* capping assays. With pppAG-RNA as the reaction substrate, GpppAG-RNA was synthesized by replacing SAM and ScriptCap 2’-O-methyltransferase with RNase-free H_2_O, m7GpppAG-RNA (Cap 0) was synthesized by replacing ScriptCap 2’-O-methyltransferase with the same amount of RNase-free H_2_O, and m7GpppAmG-RNA (Cap 1) was synthesized according to the instructions. Various capped RNAs were transfected into DEF and BHK-21 cells precultured in 24-well plates or 12-well plates using Lipofectamine MessengerMAX™ Reagent (Invitrogen). At 4 h post-transfection (hpt), the cells from 24-well plates were harvested and lysed, after which the activities of RLuc were detected using a *Renilla* Luciferase Assay System (Promega). Meanwhile, the cells in the 12-well plates were collected, and total RNA was isolated using RNAiso Plus. Reverse transcription (RT)-qPCR that detected the RLuc gene was used to measure the copy numbers of reporter RNAs. Primers were listed in **Supplementary Table S4**.

### Statistical Analysis

The significance of the observed differences was assessed using One-way ANOVA and two-way ANOVA test with GraphPad Prism 8.0. Differences were considered significant when P-values < 0.05. Data are expressed as mean ± standard deviation (SD).

## Results

### N-7 and 2’-O Methylations of WT and Mutant TMUV MTase

**Figure 1:**
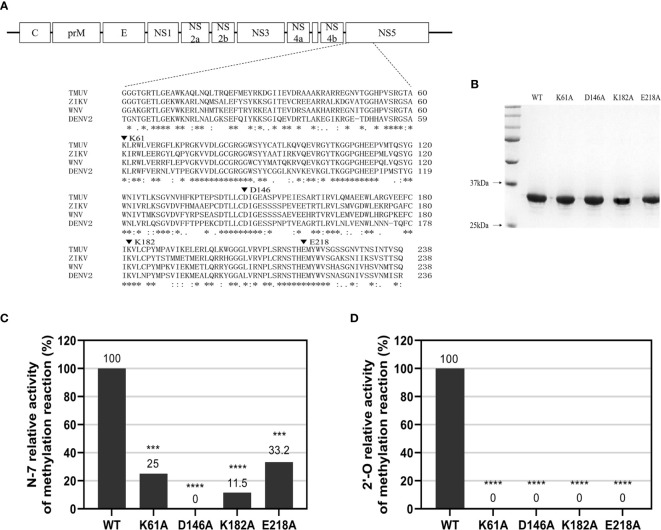
N-7 and 2’-O cap methylations of the viral RNA by WT and mutant TMUV MTase. **(A)** Sequence alignment between TMUV MTase and several other flavivirus MTase proteins. The MTase sequences of TMUV, ZIKV, WNV, and DENV2 are derived from GenBank accession numbers AIU44176.1, YP_002790881.1, NP_776022.1, and 5ZQK_A, respectively. The conserved K61-D146-K182-E218 tetrad residues mutated in this study are indicated by inverted triangle symbol. **(B)** Analysis of recombinant TMUV MTase proteins on 12% SDS-PAGE gels. **(C, D)** Effects of K61-D146-K182-E218 mutations on N-7 and 2’-O methylations. Recombinant MTase proteins were assayed for GpppAG-RNA→m7GpppAG-RNA and m7GpppAG-RNA→m7GpppAmG-RNA conversions to indicate N-7 **(C)** and 2’-O **(D)** methylations, respectively. The indicated RNA substrates were treated with recombinant MTase in the presence of SAM, digested with nuclease P1 (Sigma), and analyzed by thin-layer chromatography (TLC) plates. The G*pppAG, m7G*pppAG, and m7G*pppAmG products were measured by scintillation counting. The methylation activity of the mutant MTase was compared with that of the WT MTase (set as 100%). Each of these was conducted with three parallel replicates. ****P < 0.0001; ***P < 0.001 (one-way ANOVA).

We performed sequence alignment (**Figure 1A**) between TMUV and several flavivirus MTase proteins indicating that K-D-K-E motif is highly conserved among flaviviruses. To determine N-7 and 2’-O methylations of WT and mutant TMUV MTase, a recombinant WT and four single mutant MTase proteins, containing Ala-substitution at the K-D-K-E catalytic tetrad (K61A, D146A, K182A and E218A) and representing the N-terminal 300 amino acids of the TMUV NS5 (strain CQW1), were prepared. SDS-PAGE analysis showed that the purified proteins were pure with the expected molecular mass of 33 kDa (**Figure 1B**). Substrates G*pppAG- and m7G*pppAG-RNA, representing the first 192 nucleotides of the 5’ end of the TMUV genome, were incubated with the recombinant MTase proteins in the presence of SAM. For N-7 methylation (GpppAG→m7GpppAG), the K61A, K182A and E218A mutants retained 25%, 11.5% and 33.2% of the WT N-7 MTase activity, respectively, whereas D146A mutant completely lost the N-7 MTase activity (**Figure 1C** and **Supplementary Table S5**), showing that only D146 is essential for N-7 methylation. For 2’-O methylation (m7GpppAG→m7GpppAmG), All mutants abolished 2’-O MTase activity, demonstrating that the K-D-K-E catalytic tetrad is essential for the 2’-O methylation (**Figure 1D** and **Supplementary Table S6**).

### Recovery and Characterization of K-D-K-E Mutant TMUV

**Figure 2:**
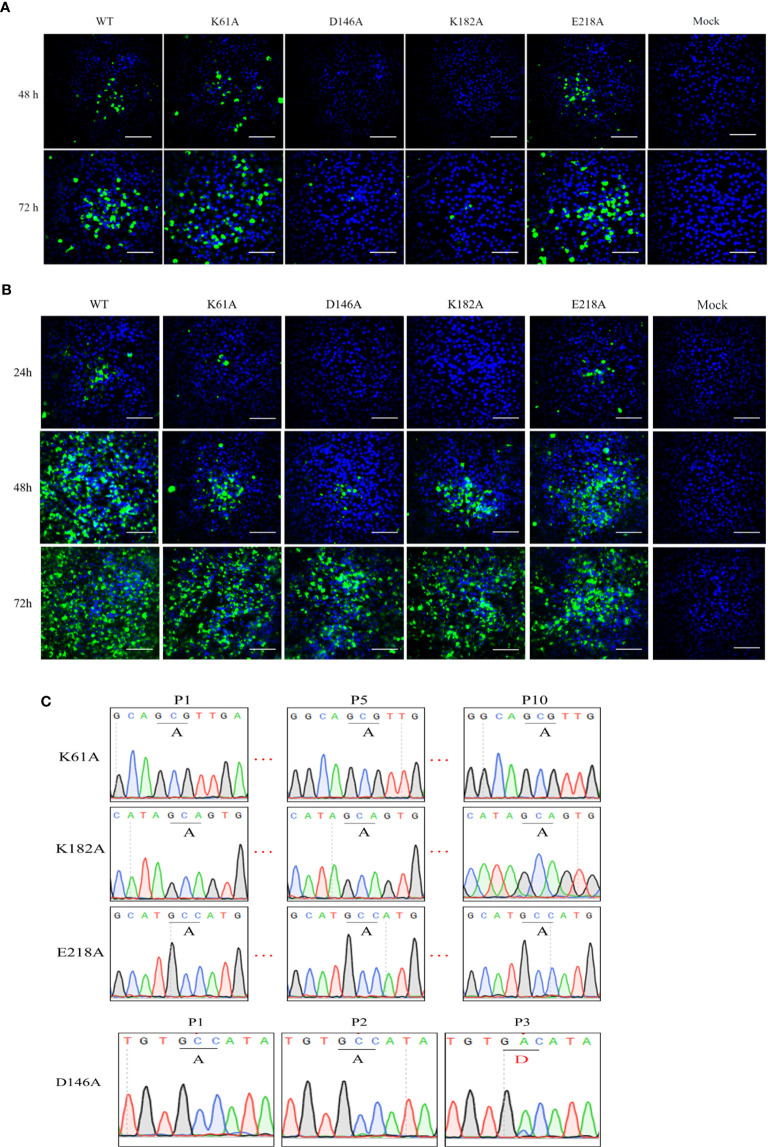
Recovery and characterization of K-D-K-E mutant TMUV. **(A, B)** BHK-21 cells were transfected with equal amounts of genome-length RNA transcripts **(A)** or infected with passage 1 (P1) viruses **(B)** and subjected to IFA using anti-TMUV E protein MAb at the indicated time. **(C)** The genomes of different passages of viruses were sequenced to analyze genetic stability.

To investigate the phenotype of K-D-K-E mutant TMUV, we introduced an alanine substitutions in the K-D-K-E motif into the full-length infectious clone of TMUV pACYC-TMUV (35), and obtained four TMUVs with single mutation of K61A, D146A, K182A or E218A. Viral E protein-positive cells were observed in BHK-21 cells transfected with genome-length RNAs obtained by *in vitro* transcription (**Figure 2A**) or infected with passage 1 (P1) viruses (**Figure 2B**). The numbers of fluorescent spots in K61A, D146A, K182A and E218A groups were significantly less than WT group (data not shown). Sequencing of the P1 viral genome confirmed that the designed K61A, D146A, K182A and E218A mutations were retained in the recovered viruses and no additional mutations were introduced (**Figure 2C**). The genetic stability analysis of the mutant viruses showed that the engineered K61A, K182A and E218A changes were retained even after 10 rounds of passage, whereas the change of D146A was restored to D146 of the wild-type virus in the third passage (**Figure 2C**). The results showed that the K61A, K182A and E218A mutant viruses were genetically stable in cell culture except for D146A virus.

### Mutant TMUVs Are Attenuated *In Vitro*

**Figure 3:**
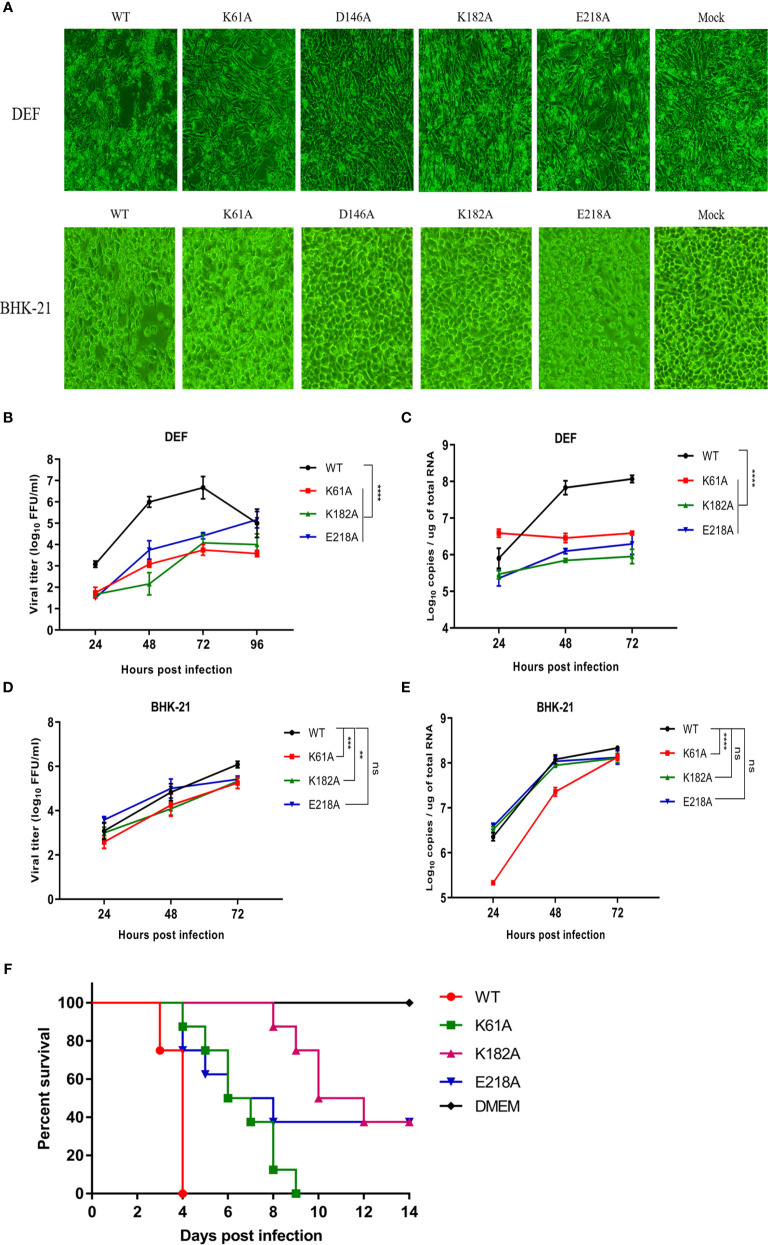
Mutant TMUVs are attenuated *in vitro*. **(A)** Cytopathic effects of WT and mutant viruses in DEF and BHK-21 cells. Cells were infected with P1 viruses at a MOI of 0.01, and the extent of cytopathic changes was observed daily. The images were taken by post-infection day 4. **(B–E)** Growth kinetics of WT and mutant viruses in DEF **(B, C)** and BHK-21 **(D, E)** cells. Cells were infected with WT and mutant viruses at a MOI of 0.01. Viral titers and RNA copy numbers were measured at indicated times using fluorescence formative unit (FFU) and absolute qPCR assays. Each of these was conducted with three parallel replicates. Bars show means ± SDs. ****P < 0.0001; ***P < 0.001; **P < 0.01 (two-way ANOVA). **(F)** Virulence determination of WT and mutant viruses in duck embryos. 9-day-old embryonated duck eggs (n = 8) were infected by allantoic-cavity inoculation with 10^4^ FFU of WT, K61A, K182A or E218A virus, respectively, while the mock-infection group received 100 ul of DMEM. ns, no significance.

We compared the cytopathic effects of WT and mutant viruses in DEF and BHK-21 cells. Cells were infected with P1 viruses at a MOI of 0.01, and the extent of cytopathic changes was observed daily. At 4 days post-infection, the D146A and K182A viruses were unable to cause CPE to DEF and BHK-21 cells, while K61A and E218A viruses could cause CPE to cells, but the damage was less severe than that caused by WT virus (**Figure 3A**). Due to the genetic instability of D146A virus, it was not involved in subsequent viral infection trials. Growth curves showed that in DEF cells, the proliferation ability of K61A, K182A and E218A viruses showed >1.5-log lower than that of WT virus at 24, 48, and 72 hpi (**Figure 3B**), and the copy numbers of K61A, K182A and E218A viruses were also >1.5-log lower than that of WT virus at 48 and 72 hpi (**Figure 3C**). In BHK-21 cells, the viral titers of K61A and K182A viruses were 1-log lower than that of WT virus at 48 and 72 hpi (**Figure 3D**). The copy numbers of K61A virus showed approximately 1-log lower than that of WT virus at 24 and 48 hpi (**Figure 3E**). Although there was no significant difference in growth kinetics between E218A virus and WT virus, the growth of E218A virus showed a trend of slowing down significantly 48 h after infection (**Figures 3D, E** **)**. Then, we investigated the virulence of K61A, K182A and E218A viruses in duck embryos. The embryos infected with WT virus all died 4 days post-infection. All the duck embryos infected with K61A virus died 9 days post-infection. Up to 14 days after infection, there were three surviving duck embryos in each group infected with K182A or E218A, and the lethal time of these mutant viruses to duck embryos was significantly delayed (**Figure 3F**). Taken together, these results indicate that K-D-K-E mutations have significantly reduced TMUV proliferation and virulence *in vitro*.

### K-D-K-E Mutations May Reduce Translation Efficiency of TMUV Luciferase Reporter Replicon

**Figure 4:**
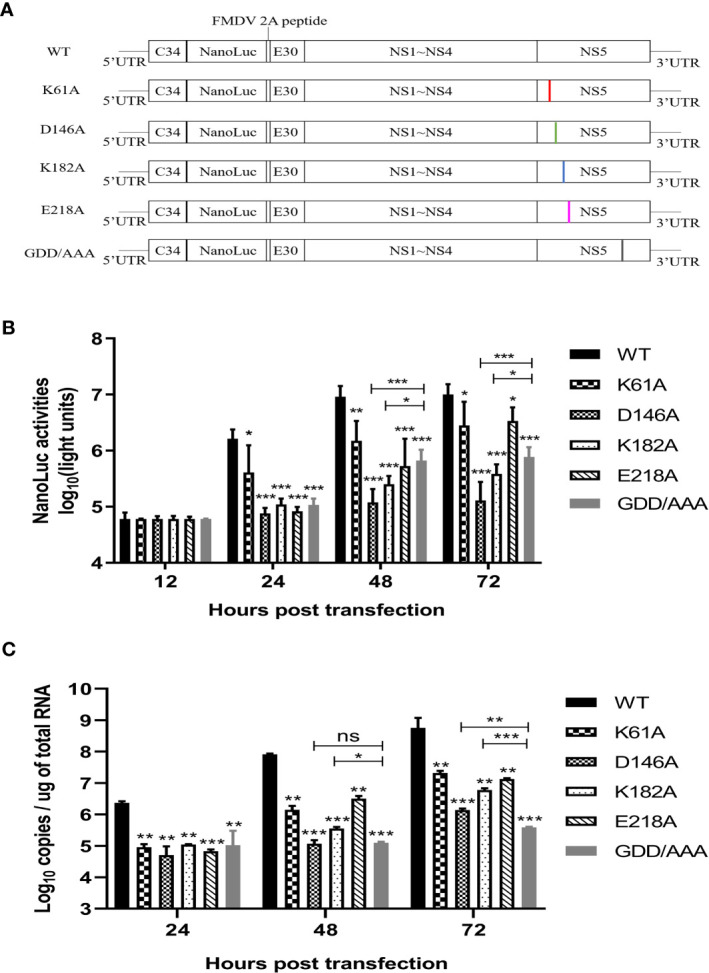
K-D-K-E mutations may reduce translation efficiency of TMUV luciferase reporter replicon. **(A)** schematic diagram presentation of WT and mutant TMUV replicons. **(B)** NLuc activity assay. BHK-21 cells were transfected with equal amounts of WT and mutant replicons. At 12, 24, 48 and 72 h post-transfection, the cells were lysed, and NLuc activities were measured. Each of these was conducted with three parallel replicates. Bars show means ± SDs. ***P < 0.001; **P < 0.01; *P < 0.05 (two-way ANOVA) **(C)** Copy numbers of replicons were determined by absolute qPCR. Each of these was conducted with three parallel replicates. Bars show means ± SDs. ***P < 0.001; **P < 0.01; *P < 0.05 (two-way ANOVA). ns, no significance.

Then, we engineered the K61A, D146A, K182A or E218A mutations into a Nano-Glo luciferase reporter TMUV replicon with CMV promoter according to the schematic diagram in **Figure 4A**. Replication was monitored by Nano-Glo luciferase (NLuc) activity encoded by TMUV replicon. After transfection of BHK-21 cells with equal amounts of replicons, the K61A, D146A, K182A, and E218A mutant replicons produced lower levels of NLuc activity than WT replicon at 24, 48 and 72 h post-transfection, showing a level-dependent reduction in N-7 methylation (**Figure 4B**). A parallel experiment was performed. The copy numbers of replicons were determined by absolute qPCR that detected NS3 gene. The copy numbers of K61A, D146A, K182A, and E218A mutant replicons were considerably lower than that of WT replicon at 24, 48 and 72 h post-transfection, which also showed a decrease in the dependence of N-7 methylation levels (**Figure 4C**). These results indicate that K61A, D146A, K182A and E218A mutations lead to a decrease in replicon replication capacity. Interestingly, we found that the copy numbers of D146A and K182A replicons were significantly higher than or equal to that of GDD/AAA replicon (the replicon harbors a GDD-to-AAA mutation in the active site of the NS5 RNA polymerase that is conserved among flaviviruses, which leads to loss of RNA polymerase activity and blocked RNA synthesis of the replicon, so it is generally used as a negative control group (37, 38).) at 48 and 72 h post-transfection (**Figure 4C**), but the NLuc activities produced by D146A and K182A replicons were significantly lower than that produced by the GDD/AAA replicon (**Figure 4B**), indicating that D146A and K182A mutations inhibit translation of the replicon. This result suggests that the K-D-K-E mutations may inhibit viral translation.

### K-D-K-E Mutations Inhibit Efficient Expression of Viral Proteins

**Figure 5:**
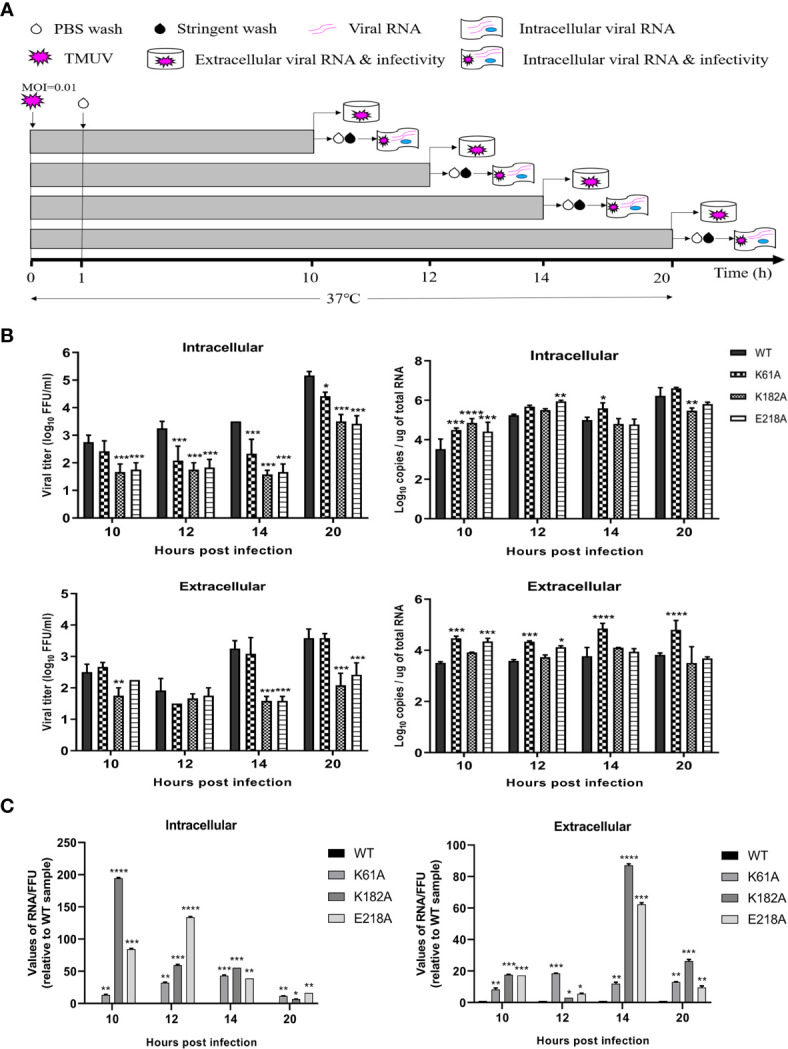
K61A, K182A and E218A mutations inhibit efficient expression of viral proteins. **(A)** Flowchart of monitoring of TMUV infection. **(B)** Quantification of intra- and extracellular infectious virions and viral RNAs at 10, 12, 14 and 20 hpi. At given time points, intracellular and extracellular infectious virions were measured by FFU assay and intracellular and extracellular viral RNAs were quantified by absolute qPCR. **(C)** The ratio of RNA/FFU. Each of these was conducted with three parallel replicates. Bars show means ± SDs. ****P < 0.0001; ***P < 0.001; **P < 0.01; *P < 0.05 (two-way ANOVA).

In the life cycle of flavivirus, the protein expression period of flavivirus is generally 10-20 h after infection, and the most vigorous period of virus translation is concentrated in 10-14 hpi (36). To identify whether the K-D-K-E mutations inhibit the effective expression of viral proteins, we used recombinant K61A, K182A and E218A viruses to examine the effect of these mutations on protein expression period. **Figure 5A** depicts the experimental flowchart. The entire duration of infection was limited to 20 h to avoid multiple rounds of infection. DEF cells were infected with 0.01 MOI of WT, K61A, K182A or E218A viruse at 37°C for 1 h. cells were washed with PBS to remove unadsorbed viruses. After additional 9, 11, 13 and 19 h of incubation at 37°C, we measured intracellular and extracellular virions by FFU assay and intracellular and extracellular viral RNAs by qPCR that detected E gene. Compared with the WT, the K61A mutant at 10, 12, 14 and 20 hpi produced fewer intracellular virions and the equal amounts of extracellular virions, but more viral RNAs both intracellular and extracellular (**Figure 5B**). In the K182A, fewer intracellular and extracellular virions were detected at 10, 12, 14 and 20 hpi in compared to the WT, and almost the equal amounts of viral RNAs were produced both intracellular and extracellular for K182A and WT viruses, excepting that the K182A intracellular viral RNAs were slightly higher or lower than that of WT at 10 and 20 hpi (**Figure 5B**). Compared with the WT, the E218A mutation produced fewer intracellular virions at 10, 12, 14 and 20 hpi, and fewer extracellular virions at 14 and 20 hpi, but more intracellular and extracellular viral RNAs at 10 and 12 hpi (**Figure 5B**). An RNA/FFU ratio reflects a higher amount of intracellular or extracellular RNA and lower intracellular or extracellular infectious virions in mutant viruses group indicating that the K61A, K182A and E218A mutations reduced the efficiency of RNA packaging into infectious virions (**Figure 5C**). Overall, K61A, K182A and E218A mutations produced fewer infectious virions but more or equal amounts of viral RNAs in compared to the WT, indicating that the K61A, K182A and E218A mutations increase or do not affect viral RNA synthesis but significantly inhibit efficient expression of viral proteins.

### Defect in N-7 Methylation Leads to Translational Suppression of Mutant Viruses

**Figure 6:**
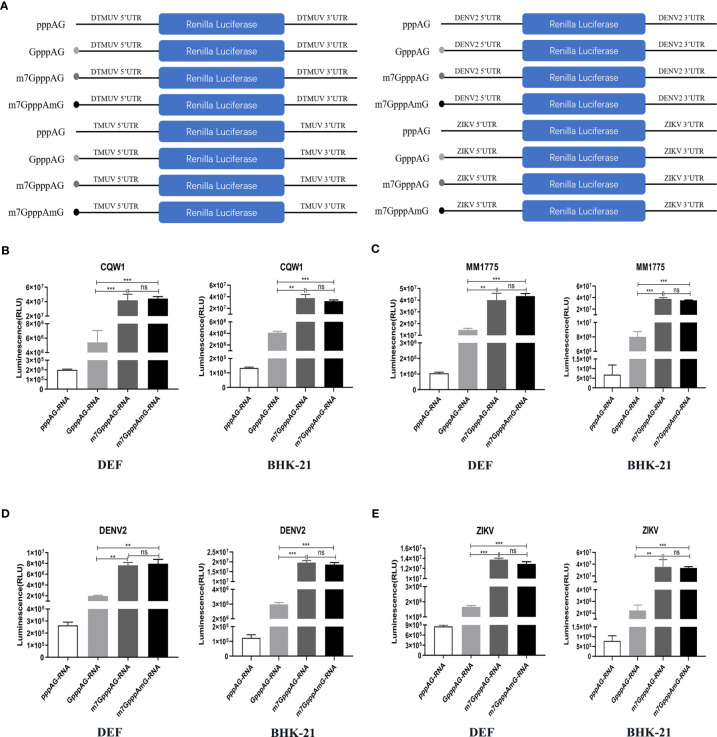
Defect in N-7 methylation leads to translational suppression of mutant viruses. **(A)** The construction schematic of the RNA translation templates with the different components of cap structures for TMUV, DENV2, and ZIKV. **(B–E)** DEF and BHK-21 Cells were transfected with equal amounts of RNAs. The RLuc activities of different RNAs of TMUV strain CQW1 **(B)**, TMUV strain MM1775 **(C)**, DENV2 **(D)** and ZIKV **(E)** were measured at 4 h post-transfection. Each of these was conducted with three parallel replicates. Bars show means ± SDs. ***P < 0.001; **P < 0.01 (one-way ANOVA). ns, no significance.

To further identify whether K-D-K-E mutations inhibit viral translation and which methylation defects are responsible for translational suppression of mutant viruses, we constructed a variety of RNA translation templates with the different components of cap structures, containing the coding sequence of RLuc flanked by the 5’UTR and 3’UTR of flavivirus. **Figure 6A** shows the construction schematic of the RNA translation templates. At 4 h after transfection of DEF and BHK-21 cells with equal amounts of RNAs, cells were used to determine RLuc activity using a *Renilla* Luciferase Assay System. In DEF and BHK-21 cells, The RLuc activities of m7GpppAG-RNA (Cap 0) and m7GpppAmG-RNA (Cap 1) of duck TMUV (strain CQW1, NCBI accession no. KM233707.1) and mosquito-borne TMUV (strain MM1775, NCBI accession no. MH414569) were comparable but significantly higher than that of GpppAG-RNA (>4-fold) and much higher than that of uncapped pppAG-RNA (>40-fold) (**Figures 6B, C** **)**. Similar results were observed in DENV2 and ZIKV RNA translation template trials. The RLuc activities of m7GpppAG-RNA and m7GpppAmG-RNA of DENV2 (NCBI accession no. AF038403.1) and ZIKV (NCBI accession no. KU365778.1) were comparable but over 4-fold higher than that of GpppAG-RNA and over 15-fold higher than that of uncapped pppAG-RNA in both types of cells (**Figures 6D, E** **)**. Based on the fact that a significant decrease in N-7 methylation activity led to the production of large amounts of GpppAG-RNA (**Figure 1D**), these results suggest that K-D-K-E mutations inhibit TMUV RNA translation and defect in N-7 methylation is a key factor leading to translation inhibition of K-D-K-E mutants. Furthermore, these results indicate that the capping and N-7 methylation have positive effects on translation of flaviviruses, whereas 2’-O methylation has no effect on translation of flaviviruses. Of note, uncapped pppAG-RNA detected a significantly higher RLuc fluorescence value of 10^5^-10^6^ than the background fluorescence value of 10^2^ (data not shown), suggesting that uncapped pppAG-RNA can be translated and a non-classical cap-independent translation mechanism may exist for flaviviruses (**Figures 6B–E**). To evaluate the transfection stability of translation template RNAs, a parallel experiment was performed and transfected cells were used for total RNA extraction at 4 h post-transfection. The RNA copies were determined by absolute qPCR that detected RLuc gene. **Supplementary Figure S1** showed that the amounts of different reporter RNAs transfected was almost equal.

### Mutant TMUVs Induce a Higher Innate Immune Response

**Figure 7:**
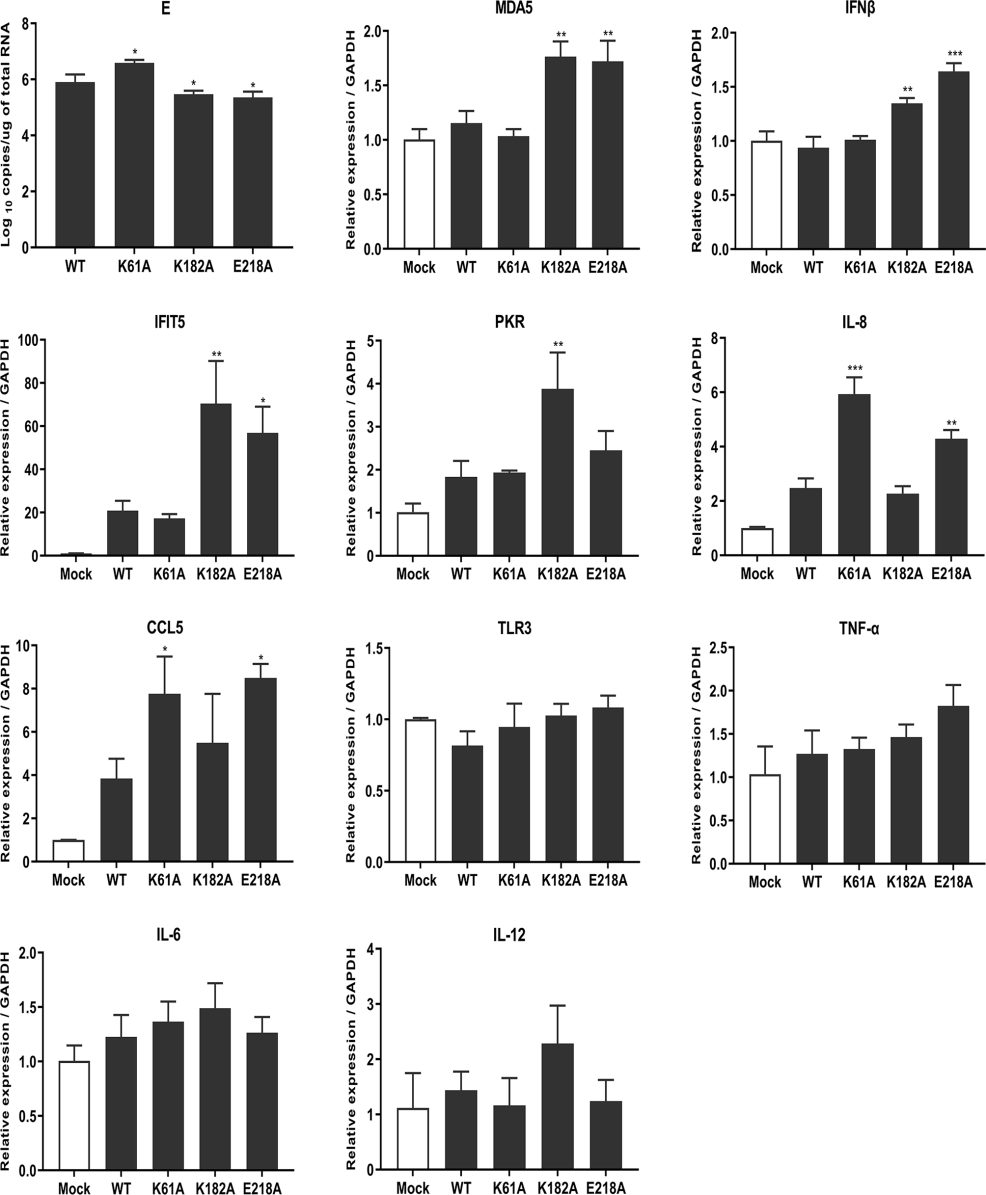
Mutant TMUVs induce a higher innate immune response. DEF cells were infected with 0.01 MOI of WT, K61A, K182A and E218A viruses, respectively. At 24 hpi, cells were harvested and transcription levels of cytokines were measured by qPCR. Each of these was conducted with three parallel replicates. Bars show means ± SDs. ***P < 0.001; **P < 0.01; *P < 0.05 (one-way ANOVA).

RNA cap methylation has been shown to be important for innate immune evasion. To investigate whether MTase-deficient TMUVs induce higher innate immune response, we measured the transcription levels of a number of cytokines including host immune sensors, type I interferons, chemokines, inflammatory cytokines using qPCR after viral infection. DEF cells were infected with 0.01 MOI WT, K61A, K182A and E218A viruses, respectively. At 24 hpi, cells were harvested for total RNA extraction and transcription levels of cytokines were measured. Compared with WT, K182A virus induced higher expression of host immune recognition sensor MDA5 and downstream IFNβ as well as Interferon-stimulated genes (ISGs) IFIT5 and PKR, and E218A virus significantly stimulated expression of MDA5, IFNβ, IFIT5, IL-8 and CCL5 genes. Interestingly, K61A virus did not activate the expression of MDA5 and IFNβ as well as IFIT5 and PKR genes, but significantly stimulated the expression of chemokines IL-8 and CCL5 (**Figure 7**). Uniformly, TLR3, TNF-α, IL-6 and IL-12 genes had no statistical difference in the quantities induced when comparing each of the mutants to WT. Overall, these results suggest that MTase-deficient TMUVs induce a higher innate immune response that is not dependent solely on the type I interferon response.

## Discussion

The aim of this study was to investigate the function of TMUV MTase and its important role in the viral life cycle and virus-host interaction. A K-D-K-E catalytic tetrad localized to the N-terminal MTase region of the NS5 protein is highly conserved in flavivirus members and is responsible for both N-7 and 2’-O methylations. This study was performed with K-D-K-E catalytic tetrad as the target. We determined the *in vitro* activity of TMUV MTase and investigated the influence of K-D-K-E motif mutations on methylation activities and viral biological characteristics. Further study of the molecular mechanisms revealed the mechanism by which the mutated virus attenuated. Ultimately, we found that MTase-deficient TMUV was attenuated due to suppression of viral RNA translation and induction of higher innate immunity. Furthermore, we found that the defect of N-7 methylation may be responsible for the inhibition of mutant viral RNA translation, whereas deletion of 2’-O methylation does not affect mutant viral RNA translation. These findings have revealed that the 5’ cap methylation of the flavivirus genome plays an important role in viral translation, proliferation, and escape from host immune responses. The discovery that uncapped RNA can be translated suggests that there may be a cap-independent nonclassical translation mechanism for flaviviruses. These findings provide a theoretical basis for the study of the translation mechanism of flavivirus.

In the present study, *in vitro* activity of TMUV MTase was determined using isotope labelling that is previously reported by others (16, 28, 29). TMUV K-D-K-E catalytic tetrad is essential for the 2’-O methylation, which is consistent with the results previously reported by other flaviviruses (16, 19). Interestingly, A slight difference was reported by Dong et al. that DENV4 K181A mutation still retained 6% of WT 2’-O methylation activity (27). For N-7 methylation, The TMUV D146A mutation completely lost the N-7 methylation activity, whereas mutations of K61A, K182A and E218A reduced N-7 methylation activity to 25%, 11.5% and 33.2% of the WT level, respectively. Similarly, the D146A substitution in WNV and DENV4 also completely abolished N-7 methylation activity. Whereas WNV K61A, K182A, and E218A substitutions retained 22%, 7%, and 16% of WT N-7 methylation activity, respectively. DENV4 K61A, K182A, and E218A substitutions retained 34%, 16%, and 59% of WT N-7 methylation activity, respectively (16, 27). These results indicate the plasticity of the role of the K-D-K-E catalytic tetrad in the methylation activities among various flaviviruses.

Previous reports suggested that the D146A mutation prevents infection/propagation of DENV and WNV (19, 27). However, TMUV D146A mutation did not prevent viral infection/propagation. IFA assay showed that positive-cells could be observed in BHK-21 cells transfected with genome-length D146A RNA or infected with P1 D146A virus. Complete genome sequencing of P1 generation D146A virus showed that the engineered mutation was retained without additional mutations. This is different from the characterization caused by the D146A mutation in DENV and WNV. However, there are certain commonalities. Although the TMUV D146A virus was rescued from RNA-transfected cells, it was unstable and reverted to WT virus in passage to the third generation. A similar but different phenomenon was observed during the rescue of WNV D146A virus. Continuous culturing of the WNV D146A RNA-transfected cells began to produce detectable virus and displayed a plaque size similar to that of the WT virus on day 8 post-transfection. Sequencing of the recovered virus showed the mutated D146A was reversed to the wild-type D residue (19). In contrast, TMUV K61A, K182A and E218A mutants were stable, which was consistent with the findings of other flaviviruses. Then, we showed that MTase-deficient TMUVs were attenuated *in vitro*. Replicon results showed that mutations of K61A, D146A, K182A and E218A significantly reduced TMUV replicon replication. K61A, K182A and E218A mutations delayed the cytopathic effect, and decreased the rate of viral proliferation on cells, and reduced the mortality of duck embryos. The results are similar to that previously observed for the MTase mutant flaviviruses. DENV, WNV, and JEV E218A mutants were attenuated *in vivo* and *in vitro* and protected the mice against lethal challenge with virulent virus strains (19, 28, 29). These results suggest that 2’-O unmethylated flaviviruses may be a potential choice for vaccine design.

What causes the attenuated virulence of MTase-deficient viruses? Many previous studies have shown that 2’-O unmethylated WNV, JEV, and DENV showed high sensitivity to the antiviral effects of IFN and IFITs (22, 28, 29). Züst et al. demonstrated that human and mouse coronavirus mutants lacking 2’-O MTase activity induced higher expression of type I interferon and were highly sensitive to type I interferon (12). And 2’-O unmethylated DENV also significantly induced an earlier innate immune response that is not dependent on a functional type I interferon response (39). In addition, 2’-O unmethylated non-self RNAs are more readily recognized by RIG-I, MDA5, and IFITs proteins to perform antiviral functions (12, 13, 21, 24, 40). In summary, RNA viruses with 2’-O MTase deficiency are more readily recognized by the host immune system and elicit an antiviral response. As expected, we found that MTase-deficient TMUV induced a higher innate immune response that was not dependent solely on the type I interferon response. There were differences in cytokines induced by the mutant viruses, which may be due to differences in N-7 methylation activity. Since previous studies have shown that the cap structure of GpppAG-RNA is also more readily recognized by RIG-I and IFITs proteins (21, 40), it is speculated that varying degrees of N-7 methylation may be related to the type of innate immune response activation, but further experiments are needed to identify. In addition to causing immune activation, we found for the first time that K-D-K-E mutations inhibit effective expression of viral proteins. Further experimental studies inferred that the translation inhibition of the mutant viruses was due to a defect in N-7 methylation rather than 2’-O methylation. Although the K61A, K182A, and E218A mutations did not completely abolish N-7 methylation activity, a significant reduction in N-7 methylation activity led to the production of N-7 unmethylated RNAs, which reduced the translation efficiency of the mutant viruses. In the DENV and ZIKV translation templates experiments, it was also found that a defect in N-7 methylation but not in 2’-O methylation resulted in RNA translation inhibition, suggesting that N-7 methylation plays a key role in cap-dependent translation for flaviviruses. The uncapped RNA, pppAG-RNA, was capable of translation, suggesting that there may be a cap-independent non-classical translation mechanism for flaviviruses. In 2006, Edgil et al. reported that DENV utilizes a novel strategy for translation initiation, which was not regulated by the internal ribosomal entry site but was controlled by the interaction of the 5’ and 3’ terminals (41). However, recent work published by Song et al. reported that the 5’UTR of DENV and ZIKV harbor internal ribosomal entry site functions (42). Combined with our results and these previously reported, it is certain that a non-classical translation mechanism for flaviviruses does exist, but its true nature remains controversial and needs further investigation. The study of the cap-independent non-classical translation mechanism of flaviviruses will help us to better understand flaviviruses and develop novel antiviral strategies.

In conclusion, we demonstrate that MTase-deficient TMUVs are attenuated due to suppression of viral RNA translation and induction of a higher innate immune response. And our data reveal an important role for cap-methylation in viral proliferation, translation, and escape innate immunity. Since MTase-deficient TMUVs are significantly attenuated, MTase may be an attractive target for the development of live attenuated vaccine and antiviral drug. This study just provides a scientific theoretical basis for the development of live attenuated vaccine and antiviral drug.

## Data Availability Statement

The original contributions presented in the study are included in the article/**Supplementary Material**. Further inquiries can be directed to the corresponding authors.

## Author Contributions

XW designed and performed the experiments, and wrote the manuscript. RJ conceived and supervised the study. YZ participated in some experiments. AC and MW provided substantial contributions to improve the content of the article. Other authors contributed to analysis the experimental data. All authors contributed to the article and approved the submitted version.

## Funding

This work was supported by the National Natural Science Foundation of China (31872475), Sichuan Veterinary Medicine and Drug Innovation Group of China Agricultural Research System (CARS-SVDIP), and China Agricultural Research System (CARS-42-17).

## Conflict of Interest

The authors declare that the research was conducted in the absence of any commercial or financial relationships that could be construed as a potential conflict of interest.

## Publisher’s Note

All claims expressed in this article are solely those of the authors and do not necessarily represent those of their affiliated organizations, or those of the publisher, the editors and the reviewers. Any product that may be evaluated in this article, or claim that may be made by its manufacturer, is not guaranteed or endorsed by the publisher.

## Supplementary Material

Supplementary Figure 1 Stability analysis of the transfection of translation template RNAs. Cells transfected with RNA reporters were lysed at 4 h post-transfection, and total RNA was extracted. Absolute qPCR that detected RLuc gene were used to measure the RNA copy numbers. Each of these was conducted with three parallel replicates. Bars show means ± SDs (one-way ANOVA).
