# Supplementary figures and images for: Methyltransferase-Deficient Avian Flaviviruses Are Attenuated Due to Suppression of Viral RNA Translation and Induction of a Higher Innate Immunity
Source: Front Immunol. 2021 Oct 6;12:751688. doi: 10.3389/fimmu.2021.751688 (PMC8526935; doi:10.3389/fimmu.2021.751688)

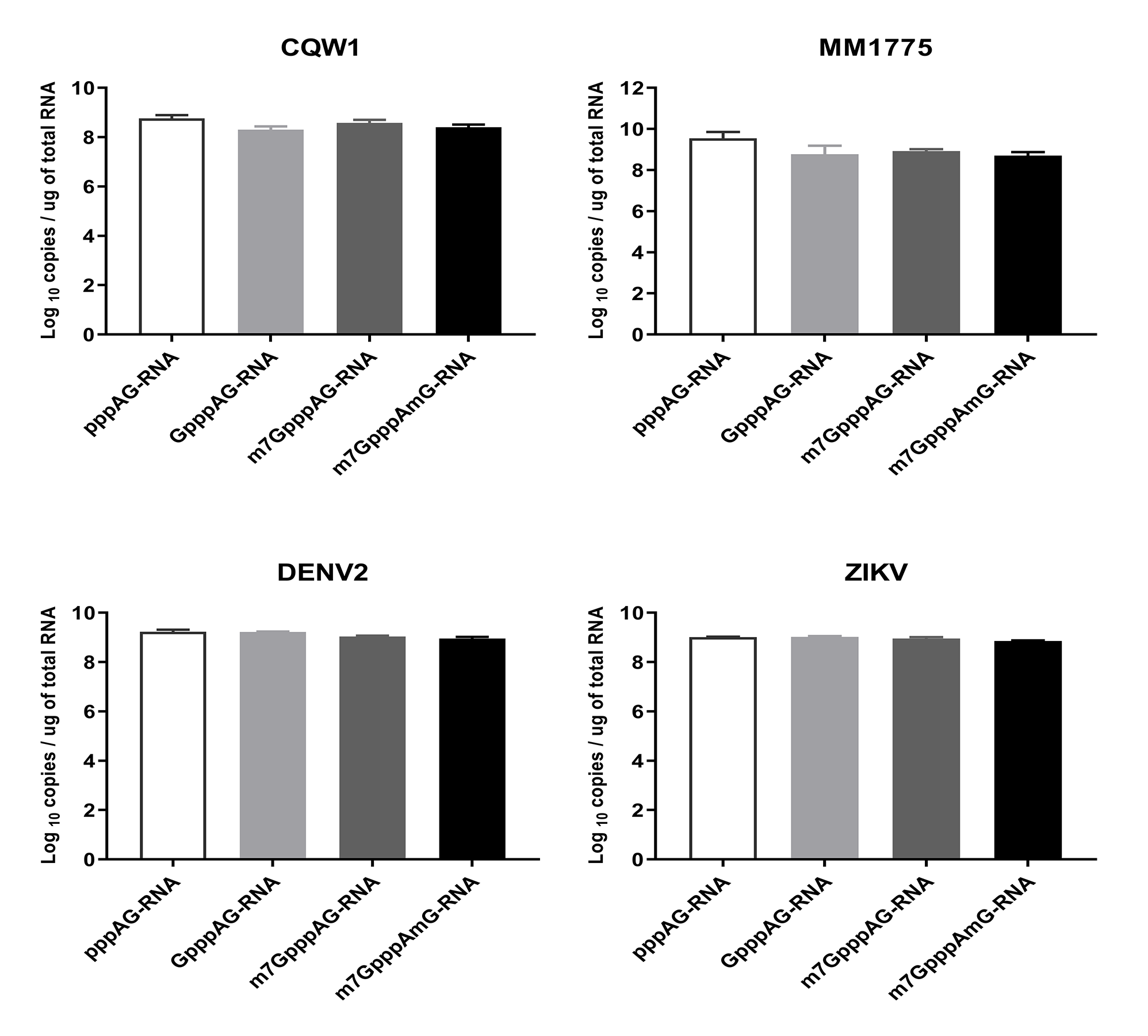

Supplement: Supplementary Figure 1 — Stability analysis of the transfection of translation template RNAs. Cells transfected with RNA reporters were lysed at 4 h post-transfection, and total RNA was extracted. Absolute qPCR that detected RLuc gene were used to measure the RNA copy numbers. Each of these was conducted with three parallel replicates. Bars show means ± SDs (one-way ANOVA). [file Image_1.tif]
