# Supplementary material for: Methyltransferase-Deficient Avian Flaviviruses Are Attenuated Due to Suppression of Viral RNA Translation and Induction of a Higher Innate Immunity
Source: Front Immunol. 2021 Oct 6;12:751688. doi: 10.3389/fimmu.2021.751688 (PMC8526935; doi:10.3389/fimmu.2021.751688)
Supplement: Supplementary file 2 [file DataSheet_1.doc]

**TABLE S1** Mutant primers of K-D-K-E

| Primer | Sequence (5’-3’) |
| --- | --- |
| K61A-F | TCACGAGGCACGGCAGCGTTGAGGTGG |
| K61A-R | GCTGCCGTGCCTCGTGAAACTGGGTGC |
| D146A-F | TCTGACACACTGCTATGTGCCATAGGTGAA |
| D146A-R | GCACATAGCAGTGTGTCAGATGGTTCAGTC |
| K182A-F | GTTGAAGAGTTCTGCATAGCAGTGCTTTGT |
| K182A-R | GCTATGCAGAACTCTTCAACGCCCCTAGCT |
| E218A-F | CGCGTAATTCAACGCATGCCATGTACTGGG |
| E218A-R | GGCATGCGTTGAATTACGCGAGAGTGGCAC |

**TABLE S2** Primers for amplification of PCR fragments containing K-D-K-E mutations

| Primer | Sequence (5’-3’) |
| --- | --- |
| EcoRv-F | AGACCTCCCTGTCTGGATATCGTACAAGGTCGCTGAAGCTGG |
| SbfI-R | CATAACCCTAGCTGTTCCTGCAGGTGGTTCTGGGGCTTTGGTAT |

**TABLE S3** Primers for reverse transcription and whole genome sequencing

| Primer | Sequence (5’-3’) | Description |
| --- | --- | --- |
| Sequencing 1-F | AGAAGTTCATCTGTGTGAACTTATTCC | Primers for full-length segmented amplification and sequencing of TMUV genome |
| Sequencing 1-R | CGGTACCATAATCCTCCATCTCAGC |
| Sequencing 2-F | GGAAGCGAGCACCTACCACAAT |
| Sequencing 2-R | CTGGGCACTCTCTAGTTTTTGGTCC |
| Sequencing 3-F | GGAGAGCTCACGTACGGATGGAAGA |
| Sequencing 3-R | TCTATCCCCACTATTCTGAGTCCTG |
| Sequencing 4-F | CTTGGCGTTGCGTTAGCACTCAT |
| Sequencing 4-R | CCTTTCAGTGCTTCCGCTATTTCAG |
| Sequencing 5-F | AAAAGGCAACTGACAGTTCTGGACC |
| Sequencing 5-R | GGCTGGGACTTCTGCTATCCATAAC |
| Sequencing 6-F | TGACTACAGCTGAGAAAGGGAGTAG |
| Sequencing 6-R | AGCAGTGTGTCAGATGGTTCAGTCG |
| Sequencing 7-F | GTGAGAGGTTACACAAAAGGAGGGC |
| Sequencing 7-R | CTTGCAAGTGCAGTTCTCTCTCTCT |
| Sequencing 8-F | GAGAAGGTGAATAGTAACGCAGCCC |
| Sequencing 8-R | TCCTTCCTGTGGGGACCCATGAGAC |
| Sequencing 9-F | GTGGTTCCATGTCGAGACCAGGATG |
| Sequencing 9-R | AGATCCTGTGTTCTACCACCACCAG |
| CQW1-RT-1847 | CAGCTTCAAACCCTGC | Specific reverse transcription primers |
| CQW1-RT-3602 | GGCTCCAATTGCTTGG |
| CQW1-RT-5235 | GTTTGTCAATGGCGCT |
| CQW1-RT-7239 | TCATGACTGCCGAGAC |
| CQW1-RT-9060 | TCCCGAACTCTCCCAT |
| CQW1-RT-10992 | AGATCCTGTGTTCTAC |

**TABLE S4** Primers for qPCR

| Primer | Sequence (5’-3’) |
| --- | --- |
| TMUV-E-qF | AATGGCTGTGGCTTGTTTGG |
| TMUV-E-qR | GGGCGTTATCACGAATCTA |
| TMUV-NS3-qF | TAAAGAGGGAGCATACTGG |
| TMUV-NS3-qR | GCAGGGTCTGTGAAGTGA |
| RLuc-qF | CCTCGTGAAATCCCGTTAG |
| RLuc-qR | TGGCACCTTCAACAATAGC |
| Duck-GAPDH-qF | TGCTAAGCGTGTCATCATCT |
| Duck-GAPDH-qR | AGTGGTCATAAGACCCTCCA |
| Duck-MDA5-qF | GCTACAGAAGATAGAAGTGTCA |
| Duck-MDA5-qR | CAGGATCAGATCTGGTTCAG |
| Duck-TLR3-qF | GCAACCAGAACTGTGCAAAAA |
| Duck-TLR3-qR | GGTTGGTGCAGGAAGCAAA |
| Duck-IFIT5-qF | AAGCTACCTTCAAACGGGTA |
| Duck-IFIT5-qR | TCCTCCTTCAGCAAAGTCCA |
| Duck-PKR-qF | AATTCCTTGCCTTTTCATCCAA |
| Duck-PKR-qR | TTTGTTTTGTGCCATAGCTTGG |
| Duck-IFNβ-qF | TCTACAGAGCCTTGCCTGCAT |
| Duck-IFNβ-qR | TGTCGGTGTCCAAAAGGATGT |
| Duck-IL-8-qF | CCTGGTAAGGATGGGAAACG |
| Duck-IL-8-qR | CGTCAGCTTCACATCTTGAATAGA |
| Duck-CCL5-qF | CCCAGACGAAGGAGAAACCT |
| Duck-CCL5-qR | AGATGGTTGTGTCAGCTCCA |
| Duck-TNF-α-qF | CATTTGGAAGCAGCGTTTGG |
| Duck-TNF-α-qR | GGTTGTGGGACAGGGTAGGG |
| Duck-IL-6-qF | CGTGTGCGAGAACAGCATG |
| Duck-IL-6-qR | GTCTCGGAGGATGAGGTG |
| Duck-IL-12-qF | GGCTCGCACCGATAAATCTG |
| Duck-IL-12-qR | TCAGTCGGCTGGTGCTCTT |

**TABLE S5** N-7 methylation (CPM)

| Site (cm) | WT | K61A | D146A | K182A | E218A | G*pppAG | m7G*pppAG |
| --- | --- | --- | --- | --- | --- | --- | --- |
| 1 | 0 | 0 | 0 | 0 | 0 | 0 | 0 |
| 2 | 0 | 0 | 0 | 0 | 0 | 0 | 0 |
| 3 | 617 | 611 | 745 | 551 | 563 | 635 | 0 |
| 4 | 0 | 0 | 0 | 0 | 0 | 0 | 0 |
| 5 | 0 | 0 | 0 | 0 | 0 | 0 | 0 |
| 6 | 0 | 0 | 0 | 0 | 0 | 0 | 0 |
| 7 | 160.42 | 33 | 0 | 13.3 | 41 | 0 | 635 |
| 8 | 0 | 0 | 0 | 0 | 0 | 0 | 0 |
| 9 | 0 | 0 | 0 | 0 | 0 | 0 | 0 |
| 10 | 0 | 0 | 0 | 0 | 0 | 0 | 0 |
| 11 | 0 | 0 | 0 | 0 | 0 | 0 | 0 |
| 12 | 0 | 0 | 0 | 0 | 0 | 0 | 0 |

Note: 1-12cm: represents the site of the chromatography paper from bottom to top. Any CPM value less than 5 is marked as 0.

**TABLE S6** 2’-O methylation (CPM)

| Site (cm) | WT | K61A | D146A | K182A | E218A |
| --- | --- | --- | --- | --- | --- |
| 1 | 0 | 0 | 0 | 0 | 0 |
| 2 | 0 | 0 | 0 | 0 | 0 |
| 3 | 0 | 0 | 0 | 0 | 0 |
| 4 | 0 | 0 | 0 | 0 | 0 |
| 5 | 0 | 0 | 0 | 0 | 0 |
| 6 | 0 | 0 | 0 | 0 | 0 |
| 7 | 128 | 217 | 220 | 243 | 280 |
| 8 | 80 | 0 | 0 | 0 | 0 |
| 9 | 0 | 0 | 0 | 0 | 0 |
| 10 | 0 | 0 | 0 | 0 | 0 |
| 11 | 0 | 0 | 0 | 0 | 0 |
| 12 | 0 | 0 | 0 | 0 | 0 |

Note: 1-12cm: represents the site of the chromatography paper from bottom to top. Any CPM value less than 5 is marked as 0.
